# New Approach for Preparing Solid Lipid Nanoparticles with Volatile Oil-Loaded Quercetin Using the Phase-Inversion Temperature Method

**DOI:** 10.3390/pharmaceutics14101984

**Published:** 2022-09-20

**Authors:** Yotsanan Weerapol, Suwisit Manmuan, Nattaya Chaothanaphat, Sontaya Limmatvapirat, Jitnapa Sirirak, Poomipat Tamdee, Sukannika Tubtimsri

**Affiliations:** 1 Faculty of Pharmaceutical Sciences, Burapha University, Chonburi 20131, Thailand; 2 Department of Pharmaceutical Technology, Faculty of Pharmacy, Silpakorn University, Nakhon Pathom 73000, Thailand; 3 Department of Chemistry, Faculty of Science, Silpakorn University, Nakhon Pathom 73000, Thailand

**Keywords:** solid lipid nanoparticles, quercetin, phase-inversion temperature, molecular dynamics study, volatile oil

## Abstract

Quercetin (QCT), a natural flavonoid, is of research interest owing to its pharmacological properties. However, its pharmacokinetic limitations could hinder its widespread therapeutic use. Nanocarriers, especially solid lipid nanoparticles (SLNs), might overcome this constraint. This study aimed to investigate QCT-loaded SLNs prepared via a new approach using a volatile oil. The phase-inversion temperature method was used to incorporate rosemary oil (RMO) into SLNs prepared using solid lipids possessing different chemical structures. Among the solid lipids used in the formulations, trilaurin (TLR) exhibited the smallest particle size and good stability after a temperature cycling test. SLNs prepared with a ratio of RMO to TLR of 1:3 could load QCT with an entrapment efficiency of >60% and drug loading of ~2% *w*/*w*. The smallest particle size was achieved using the polyoxyethylene-hydrogenated castor oil RH40, and the particle size depended on the concentration. The drug-release profile of QCT_TLR exhibited prolonged biphasic release for >24 h. QCT_TLR was a safe formulation, as indicated by a cell viability percentage of >75% at <2% *v*/*v*. In a computer simulation, the system with RMO enabled smaller sized SLNs than those without RMO. This new discovery shows great promise for producing SLNs via the phase-inversion temperature method with incorporation of volatile oil, particularly for delivering compounds with limited water solubility.

## 1. Introduction

Quercetin (QCT) has become an attractive biomaterial in the pharmaceutical field owing to its pharmacological activities, such as anti-inflammatory, anti-oxidant, anticancer, and antibacterial activities [1]. Nonetheless, its pharmacokinetic constraints, especially its low water solubility with low permeability, are major hindrances to its usefulness in the pharmaceutical field. The nanocarrier delivery approach has advantages that might help address this limitation.

Nanocarriers are materials with particle sizes in the nano-size range of <500 nm. Due to their small size and the variety of materials applied to prepare them, nanocarriers can enhance drug efficiency and overcome drug limitations beyond the capabilities of conventional dosage forms [2]. Among these nanocarriers, lipid nanocarriers have been widely used to deliver poorly water-soluble substances because of advantages such as skin permeability enhancement in topical dosage forms [3,4]. Several reports have shown the successful utilization of lipid-based nanocarriers, such as nanoemulsions [5], liposomes [6], nanostructured lipid carriers (NLCs) [7], and solid lipid nanoparticles (SLNs) [8], for local delivery of poorly water-soluble substances.

SLNs are prepared using lipids in a solid state at room and body temperature. The solid lipid core is coated with amphiphilic surfactants, enabling drug entrapment and release with a burst effect. Sustained release is achieved by incorporating specific substances into the solid lipid core. This approach is a key strategy for obtaining a carrier with controlled drug release [9]. Similar to nanoemulsion, SLNs can be prepared using low- and high-energy emulsification methods. The high-energy method requires high consumption of energy, using an expensive mechanical tool to generate small particles through various techniques, such as microfluidization [10], high-pressure homogenization [11], or sonication [12]. In contrast, the low-energy method does not require expensive equipment, which has led to increasing interest in this method.

The phase-inversion temperature method is a low-energy method involving changes in the solubility of polyethoxylated nonionic surfactants throughout temperature changes. At a high temperature, the surfactant is changed from hydrophilic to hydrophobic and produces negative curvatures and water-swollen reverse micelles. At a specific temperature (the PIT temperature or T_PIT_), the surfactant has an affinity for both oil and water phases, resulting in zero spontaneous curvature and extremely low interfacial tension values. When the temperature decreases below T_PIT_, hydrated nonionic surfactants exhibit high-water solubility and generally form fine droplets [13]. A few studies have reported the successful fabrication of SLNs via the phase-inversion temperature method using stearic acid as the solid lipid phase. After cooling, stearic acid forms a solid core surrounding the solid surfactant shell, including Tween 60 and Span 60 [14]. Interestingly, the PIT method has been widely used for preparing nanoemulsions loaded with a volatile oil [15,16]. Previous studies have reported that the presence of volatile and fixed oils at an optimized ratio can create a small droplet size and good, stable nanoemulsions [16,17]. Other factors affecting the formation and properties of nanoemulsions have been identified and include the surfactant type and concentration. Notably, few components and a simple preparation method can produce stable nanoemulsions with a small droplet size of <100 nm. These nanoemulsions can form because of the closeness of the components within the system and the interaction between the polyoxyethylene chain of the surfactant, terpenes from the volatile oil, and the hydrocarbon chain of the fixed oil [5,18]. Therefore, the utilization of volatile oil to create small particles in SLNs is an interesting topic. Although the PIT method is widely used in the preparation of nanoemulsion-loaded volatile oil, there are limited reports on the employment of volatile oils in the preparation of SLNs using the PIT method in order to promote small particle formation.

Rosemary oil (RMO), derived from *Rosmarinus officinalis* L., is an essential oil that demonstrates anti-oxidant, anti-inflammatory, antimicrobial, fungicidal, and anticancer activities [19]. Its combination with QCT might enhance the therapeutic effect. Previous study demonstrated the incorporation of RMO into both SLNs and NLCs in a topical product with the aim of enhancing efficiency in the treatment of diseases [20]. However, there has been limited research on the utilization of RMO as an excipient to promote small-sized SLNs. In addition, the insight of this study concerning fabrication of a particle composed of RMO, triglyceride, and polyoxyethylene hydrogenated castor oil RH40 (CRH40) using molecular dynamics simulation has not been reported.

The aim of the present study was to produce SLNs loaded with QCT by incorporating RMO through the phase-inversion temperature method. The influences of the RMO:solid lipid ratio, the chemical structures of the solid lipid, and the type and concentration of surfactants were assessed by investigating the physical properties, including the particle size, the size distribution, the thermal behavior of the solid lipid and QCT, the entrapment efficiency, the drug loading and the drug release, of the prepared SLNs. Further, a molecular simulation study of carriers with and without RMO was conducted to improve our understanding of the system. Finally, an SLN formulation with RMO was examined to assess its cytotoxicity against normal fibroblast cells by performing the 3-[4,5-dimethylthiazol-2-yl]-2,5 diphenyl tetrazolium bromide (MTT) assay.

## 2. Materials and Methods

### 2.1. SLNs Production

The phase-inversion temperature method was used to prepare SLNs. To make 100 g of nano-dispersions, the following masses were used: solid lipid—i.e., 3–9 g of TLR (Lot No. FCV02-ORIT, TCI, Tokyo, Japan), tripalmitin (TPT) (Lot No. PB7NJ-PN, TCI, Tokyo, Japan), glyceryl monostearate (GMS) (Lot No. K3120189, PC Drug Center, Bangkok, Thailand), and stearic acid (STA) (Lot No. 400F190104E, PC Drug Center, Bangkok, Thailand)); 1–7 g of RMO (Lot No. 20181115, Krungthepchemi, Bangkok, Thailand); 10 g of CRH40 (Lot No. 34188068E0, PC Drug Center, Bangkok, Thailand); and 80 g of water with or without 100 mg QCT (Lot No. A0372949, Acros Organic, Geel, Belgium). A solid lipid with or without QCT was melted at 70 °C. Upon nearly reaching the set temperature, RMO was poured into melted lipid and mixed well. The water phase was also heated to 70 °C. The oil phase was poured into the water and well-homogenized using a magnetic stirrer (RCT Basic, IKA, Staufen, Germany) at 1,350 rpm for 5 min and allowed to cool to 30 °C. The SLNs containing 100% solid lipid were also prepared with the same method and composed of 10 g of solid lipid (TLR, TPT, GMS, STA), 10 g of CRH40, and 80 g of water. The obtained SLNs were comparatively investigated. RMO:solid lipid ratios of 1:1, 1:1.5, 1:2, 1:3, and 1:4 at a total concentration of 10% *w*/*w* were studied in comparison with 100% solid lipid (SLNs without RMO). Moreover, several solid lipids possessing different structures, including TLR, TPT, GMS, and STA, were selected to incorporate into the SLNs. The effects of surfactant types (CRH40, Kolliphor EL (Lot No. 04321136W0, BASF, Ludwigshafen, Germany), Tween 20 (Lot No. 47922, PC Drug Center, Bangkok, Thailand), Tween 60 (Lot No. 46602, PC Drug Center, Bangkok, Thailand), and Tween 80 (Lot No. 45445, PC Drug Center, Bangkok, Thailand)) and concentrations (2.5–20% *w*/*w*) were also investigated.

### 2.2. Particle-Size and Size Distribution Assessment

Dynamic laser light scattering (Nano–ZS, Malvern, Worcestershire, UK) was performed to assess the SLN size and size distribution. The samples were poured into a cuvette and measurements were obtained to determine the Z-average, indicating the mean droplet size, and pDI value, denoting the size distribution. The mean ± standard deviation values from three experiments were recorded.

### 2.3. Stability and Temperature Cycling Test

The stability of the SLN was evaluated by performing a temperature cycling test. Alternately, the SLNs were kept in glass bottles at 4 °C for 24 h and 45 °C for 24 h (one cycle). After a six-cycle test, the particle size and size distribution of the resulting SLNs were compared with their initial properties. The experiments were performed in triplicate.

### 2.4. Differential Scanning Calorimeter (DSC)

Differential scanning calorimetry (DSC8000, PerkinElmer, Waltham, MA, USA) was performed to determine the thermal behavior of the lipid matrices and QCT after preparing the SLNs. Before the test, the SLNs were freeze-dried (Freezone, Labconco, Kansas City, KS, Canada). The freeze-dried SLNs, QCT, and bulk lipid were accurately weighed to 3 mg in aluminum pans and sealed using a mechanical press. The samples were heated from 30 °C to 350 °C at a heating rate of 10 °C/min under a constant nitrogen flow (flow rate = 20 mL/min).

### 2.5. Entrapment Efficiency and Drug Loading

Entrapment efficiency and drug loading were evaluated via a UV–vis spectrophotometry (U-2900, Hitachi, Chiyoda-ku, Japan). The calibration curve of QCT in ethanol solution was constructed at a wavelength of 372 nm with a coefficient of 0.9997. To quantify the entrapment efficiency and drug loading, 500 mg of SLN was accurately weighed and placed into a 50 mL volumetric flask with deionized water added up to the volume mark on the flask. Then, 5 mL of the resulting dispersion was pipetted out and filtered through a 0.45 µm nylon membrane filter. The nontrapped QCT on the membrane filter was dissolved in ethanol and filtered through a 0.45 µm nylon membrane filter, and the QCT concentration was quantitatively measured using UV–vis spectroscopy at a wavelength of 372 nm. The entrapment efficiency and drug loading were calculated according to the following equations:

$$\mathrm{Entrapment}\;\mathrm{efficiency}\;(\%)=\frac{\mathrm{QTC}\;\mathrm{total}\;-\;\mathrm{QTC}\;\mathrm{filtered}\;\mathrm{residue}}{\mathrm{QTC}\;\mathrm{total}}\;\times \;100$$

$$\mathrm{Drug}\;\mathrm{loading}\;(\%)=\frac{\mathrm{Amount}\;\mathrm{of}\;\mathrm{entrapped}\;\mathrm{QCT}}{\mathrm{Amount}\;\mathrm{of}\;\mathrm{solid}\;\mathrm{lipid}\;}\;\times \;100$$

### 2.6. Drug Release

The dialysis bag method was used to investigate the in vitro release of QCT from the SLNs. This study used dialysis membranes with a molecular weight cutoff of 6000–8000 (Cellu Sep, Membrane Filtration Products, Seguin, TX, USA). To establish a satisfactory sink state, a 35%:65% *v*/*v* mixture of absolute ethanol and distilled water, respectively, was used as the release medium. QCT_TLR and QCT solutions in ethanol:water ratios of 50:50 (QCT_Sol) with 10 mg of QCT were placed in a dialysis bag, which was placed in 200 mL of the 35%:65% *v*/*v* mixture of absolute ethanol and distilled water at 37 °C with stirring at 200 rpm. A 3 mL sample was taken out and replaced with an equivalent volume of fresh dissolution medium at 15 and 30 min and 1, 2, 4, 8, 10, and 24 h. The release sample was measured using UV–vis spectroscopy at a wavelength of 372 nm.

### 2.7. Cytotoxicity

Cytotoxicity was evaluated using the MTT assay. Before performing the sample test, an 80% confluency of normal fibroblast cells (MRC-5) was trypsinized to obtain a cell suspension. Totals of 5 × 10^3^–1 × 10^4^ cells/well of MRC-5 dispersion were added to a 96-well plate. After 24 h, various samples with concentrations varying in the range from 0.0625% to 4% *v*/*v* were placed in the cell culture plates for 24 h. Samples were removed and replaced with 5 mg/mL of the MTT solution. The tested plates were covered with aluminum foil and kept in a CO_2_ incubator at 37 °C with 5% CO_2_. After 3 h, MTT solution was removed and dimethyl sulfoxide was added into the plate, and then the absorbance of the sample was measured at 570 nm using a microplate reader (FLUOstar Omega, BMG Labtech, Ortenberg, Germany). The percentages of cell viability and cytotoxicity were calculated according to the following equations:

$$\mathrm{Cell}\;\mathrm{viability}\;(\%)=100\times \frac{\mathrm{Mean}\;\mathrm{absorbance}\;\mathrm{of}\;\mathrm{treated}\;\mathrm{cell}}{\mathrm{Mean}\;\mathrm{absorbance}\;\mathrm{of}\;\mathrm{untreated}\;\mathrm{cell}}$$

Cytotoxicity (%) = 100 − Cell viability (%)

### 2.8. Molecular Structure Optimization

The initial atom coordinates of eucalyptol, camphor, and alpha-pinene were obtained from the Cambridge Crystallographic Data Center (accession code PAR: 174438, 1T87 and 176009, respectively), whereas the TLR and CRH40 models were built using GaussView06. The topology and frcmod files for all the molecules were prepared using antechamber and parmchk2 in the AMBER20 software package, respectively [21]. Moreover, the initial structures of the SLN without RMO model and the SLN with RMO model were designed based on the weight ratio of the components of each model. The details of all the models are presented in Table 1. After the molecules in each system were rearranged using GaussView06 and UCSF Chimera, the models were built and solvated with TIP3P water using the LEAP module in AmberTools with the General AMBER Force Field 2 (GAFF2) parameters. For each system, energy minimization was performed for 20,000 steps and MD simulations were performed under a periodic boundary condition using the PMEMD module in AMBER20. All the bonds covalently linked to hydrogen atoms were constrained via the SHAKE algorithm in each simulation, allowing a time step of 0.002 ps. To simulate nonbonded and long-range electrostatic interactions, the particle mesh Ewald method was applied with a cutoff distance of 12 Å. Each simulation system was heated from 0 to 310 K for 100 ps in the NVT ensemble, followed by equilibration in the NPT ensemble at 310 K for 400 ps. Finally, the systems were simulated for 200 ns in the NPT ensemble at 310 K. Trajectory analysis, including the value of the root-mean-squared deviation (RMSD) and the radius of gyration (Rg) of the system, was performed using the CPPTRAJ module. All simulations were analyzed and imaged using Visual Molecular Dynamics (VMD) [22].

### 2.9. Statistical Analysis

SPSS version 28.0 was used for all statistical analyses (IBM SPSS Statistics for Windows, IBM Corp., Armonk, NY, USA). One-way analysis of variance was used to analyze the results, which was then followed by Tukey’s test. *p* values < 0.05 were accepted as indicating statistical significance.

## 3. Results and Discussion

### 3.1. Effects of the RMO:Solid Lipid Ratio and Type of Solid Lipid on Physical Properties of SLN

**Figure 1:**
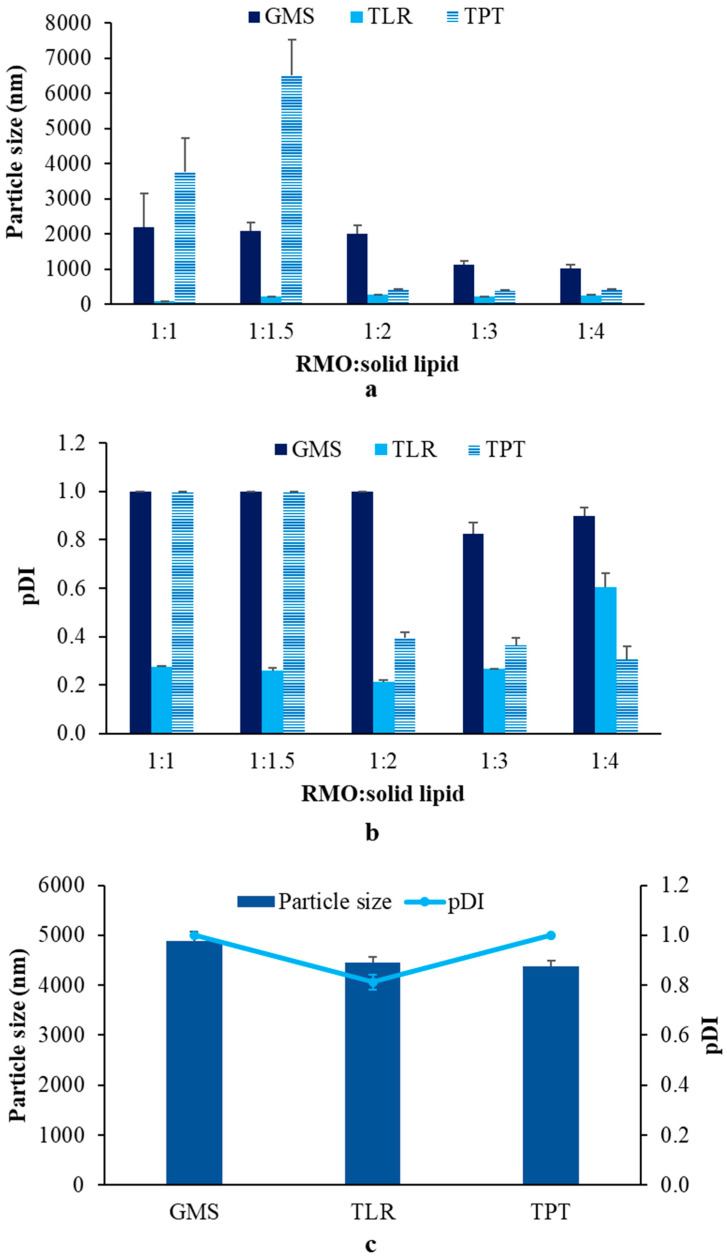
Effects of the type of solid lipid and ratio of RMO:solid lipid on particle size (**a**) and size distribution (pDI) (**b**) of SLNs, and the particle size and size distribution (pDI) of SLN prepared from 100% solid lipid (**c**).

The size and size distribution of SLNs are crucial properties affecting the stability and efficacy of the drug, and SLNs with a small size and narrow size distribution have better stability and efficacy than larger SLNs and broad size distribution. Small particle sizes and narrow size distribution are also indicators of the effectiveness of preparation methods involving several factors. In this study, the effects of the ratios and types of solid lipids on the particle size and size distribution of SLNs were compared. Figure 1 shows the sizes and size distribution of SLNs prepared using different types of solid lipids and RMO:solid lipid ratios.

Among the different solid lipids, TLR had the lowest particle size of ~85 nm and a narrow size distribution (pDI value = 0.2763 ± 0.003) when using an RMO:TLR ratio of 1:1, and the other lipids afforded SLN sizes of ~222–275 nm and pDI values less than 0.7. However, for RMO:solid lipid ratios in the range of 1:1–1:1.5, the mixed lipids were found to have a liquid form at room temperature. For TPT, a large particle size of >1000 nm with broad size distribution (pDI value = 1) was observed for SLNs prepared at the RMO/TPT ratios of 1:1 and 1:1.5, whereas the ratios of 1:2, 1:3, and 1:4 afforded SLNs with particle sizes of 423.83 ± 11.50 (pDI value = 0.3957 ± 0.0220), 394.40 ± 7.58 (pDI value = 0.3653 ± 0.0310), and 420.47 ± 6.52 nm pDI value = 0.3067 ± 0.0541), respectively. SLNs prepared using GMS exhibited large particle sizes of >1000 nm with pDI values of 0.7, indicating broad size distributions [23], at all RMO:solid lipid ratios (Figure 1a,b). STA did not form SLNs and presented in semisolid form after cooling because of its properties, which made it act as a stiffening agent when used in more than 2% *w*/*w*. Furthermore, incorporating the optimal RMO ratio into the SLNs reduced the particle size relative to the size for preparations made from 100% solid lipid, exhibiting particle sizes of 4460.67 ± 111.03, 4381 ± 108.46, and 4891 ± 180.62 nm for 100% TLR, 100% TPT, and 100% GMS, respectively, with pDI values greater than 0.7 in all formulations (Figure 1c). These results correlate with those of a previous study [12], suggesting that the composition of the oil phase and type of solid lipid are important factors influencing the SLN preparation. The presence of RMO at the optimal ratio produced the small SLNs.

Apostoloure et al. reported that the particle size of SLNs depended on the structure and melting point of the solid lipids. Smaller particle sizes resulted from lower molecular weight (MW) molecules with simple conformations rather than from higher MW molecules with more complex conformations [24]. In the present study, the particle sizes of SLNs were smaller for TLR (MW = 639) than for TPT (MW = 807.33). Nevertheless, the influence of MWs was not clearly demonstrated in the system containing GMS, although the particle size tended to decrease with decreasing molecular weight. These results suggested that other factors affected the SLN formation. Tubtimsri et al. reported that the hydrocarbon chain length of lipids directly influences the size of lipid nanocarriers when prepared using the phase-inversion temperature method. GMS has 18 carbon atoms and was the longest molecule among the solid lipids used, resulting in the largest particle size [18]. Regarding the RMO:solid lipid ratio, a ratio of <1:2 provided the largest particle size, whereas the ratios mentioned above afforded smaller sizes in all the systems, except with TLR, which might have been because of Ostwald ripening occurring immediately after preparation.

**Figure 2:**
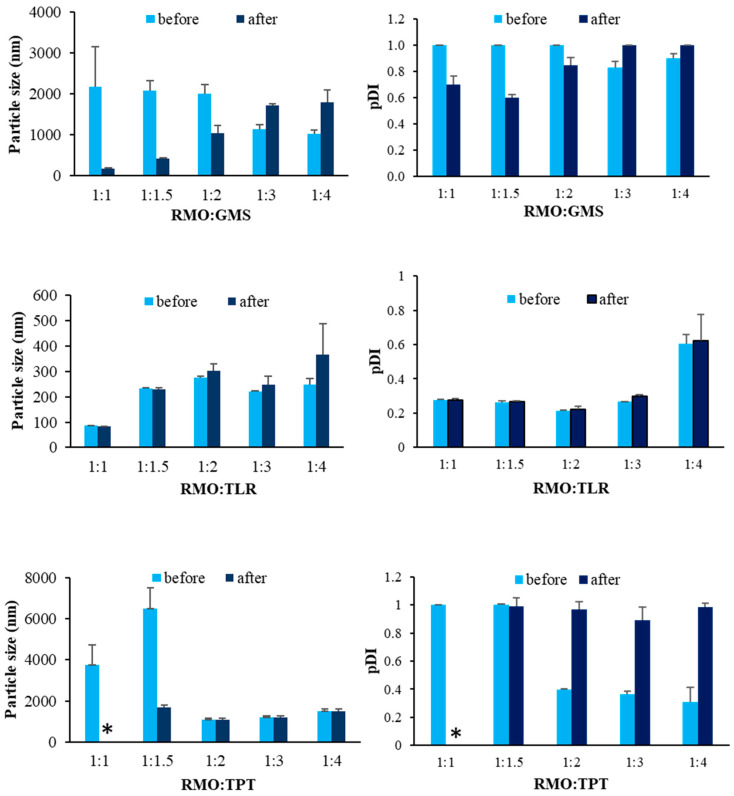
Particle size and size distribution (pDI) of SLNs prepared from different types and ratios of solid lipid before and after temperature cycling test. * represents phase separation.

Furthermore, stability testing of the SLNs was undertaken by performing a temperature cycling test, as shown in Figure 2. The RMO:TLR ratios of 1:2–1:3 exhibited the lowest alteration in the particle size and size distribution during the stability test. The particle sizes and size distributions from the other preparations were higher than those obtained initially, especially with GMS. Alteration in the particle sizes of the SLNs prepared using TPT was not clearly observed. However, the particle sizes and size distributions of SLNs prepared using GMS at a RMO:solid lipid ratio of 1:1–1:2 and TPT at a ratio of 1:1.5 tended to decrease after the test, possibly because of the insufficient amount of solid lipids used to reduce the RMO solubility (water solubility = 0.54–1767.3 mg/L at 25 °C (QSAR)) [25] in water, resulting in reduced particle sizes in accordance with Ostwald ripening [26]. The larger size after the test resulted from the increased kinetic energy and number of particles collisions, resulting in particle aggregation and larger sizes [27,28]. 

These results suggest that RMO could be used to produce stable SLNs, as shown by the unchanged particle sizes for the SLNs with the optimal concentration in the TLR system.

### 3.2. Effects of Surfactant and Concentration on Physical Properties of SLN

**Figure 3:**
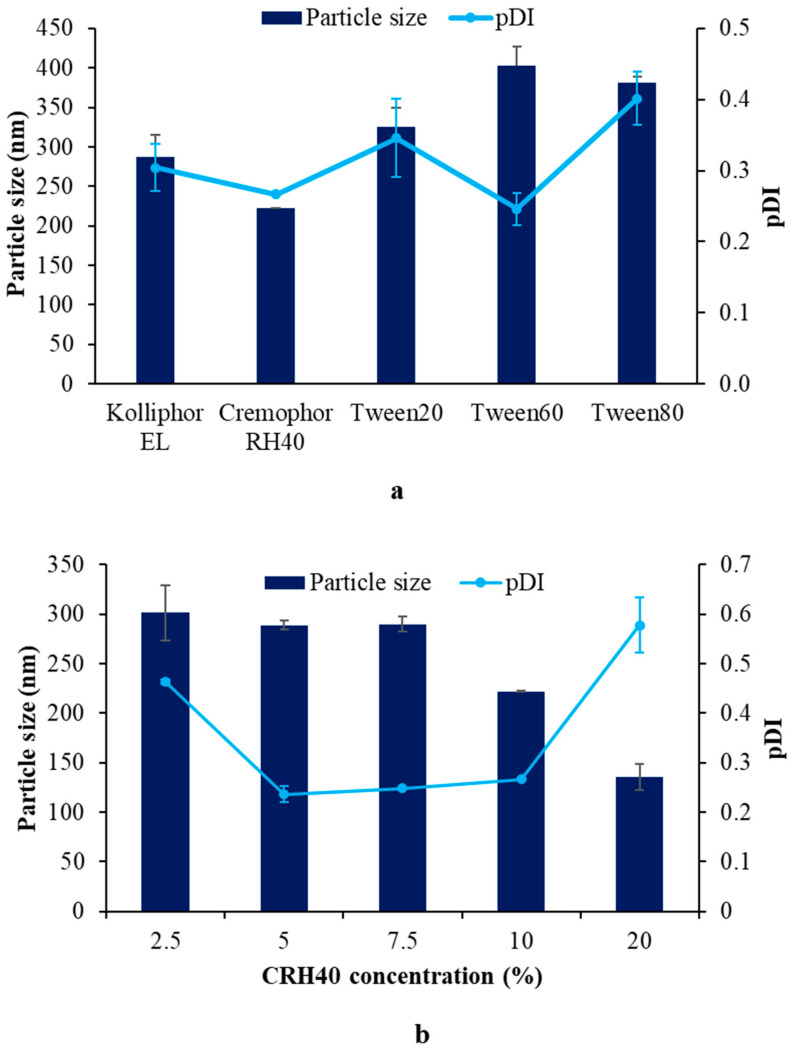
Effects of surfactant type (**a**) and concentration (**b**) on particle size and size distribution (pDI).

The type and concentration of surfactants had a significant effect on the formation of particles and the lipid carrier’s characteristics [29]. The SLNs prepared from CRH40 exhibited the lowest particle sizes, followed by those obtained from Kolliphor EL, Tween 20, Tween 80, and Tween 60 (Figure 3a). All SLNs presented pDI values less than 0.7. When considering differences between the groups of surfactants used, the particle sizes of SLNs produced by the polyoxyhydrogenated castor oil group (CRH40 and Kolliphor EL) were smaller than those obtained using the polyoxyethylene fatty acid ester group (Tween 20, 60, and 80). This result could be attributable to the polar region of the polyoxyhydrogenated castor oil group, which is larger than that of the polyoxyethylene fatty acid ester group, resulting in a high curvature in the water and oil interface that contributed to the small particle size [30]. Within each group, a considerably different particle size was observed. The particle size might be related to the HLB of the surfactants [31]. The surfactants with higher HLBs could solubilize and enhance the mixing of all compositions, resulting in smaller SLN particle sizes. The HLB of CRH40 ranged from 14 to 16, which was greater than that of Kolliphor EL (HLBs from 12 to 14) and, therefore, generated smaller particle sizes. This assumption is supported by the relation of the particle size to the HLB values: Tween 20 (HLB = 16.7) < Tween 80 (HLB = 15) < Tween 60 (HLB = 14.9). Furthermore, increasing the surfactant concentration decreased the particle size (pDI < 0.7 in all SLNs) because higher surfactant concentrations could completely cover the smaller particles with a larger overall surface area [32,33] (Figure 3b). These results suggest that the formation of SLNs prepared using the phase-inversion temperature method with RMO incorporation was associated with the surfactant properties and concentration.

### 3.3. Thermal Behavior

**Figure 4:**
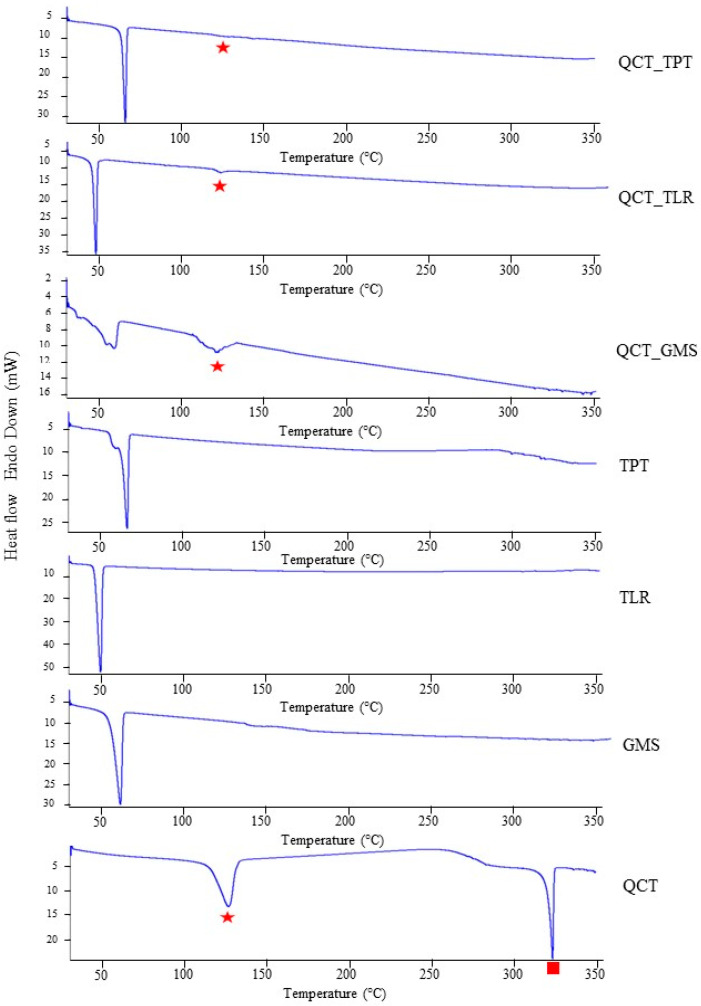
DSC thermograms of QCT, solid lipids (TLR, TPT, and GMS), and SLNs prepared using different types of solid lipids. Red square and red star represents the melting point and dehydration of QCT, respectively.

The RMO:solid lipid ratio of 1:3 was selected to load QCT because of the small particle size and good stability within each system. The thermal behaviors of the SLNs before and after loading the QCT are presented in Figure 4. QCT showed strong intensity endothermic peaks at 127.25 °C and 323.82 °C, which corresponded to dehydration and the melting point, respectively [34]. After the incorporation of QCT into the SLNs, the endothermic melting peak of QCT disappeared, which may be attributable to the solid dispersion or amorphization of QCT after its incorporation into the matrix of the SLNs. An endothermic peak due to dehydration of the QCT was observed in all the formulations, whereas the QCT_TPT had the lowest enthalpy. These results suggested that the lowest QCT loading occurred in the QCT_TPT system.

The endothermic melting point peaks of TLR, TPT, and GMS were obtained at 49.43 °C, 65.84 °C, and 60.51 °C, respectively, and all SLNs exhibited sharp endothermic melting peaks slightly lower than those of their solid lipids (QCT_TLR = 47.37 °C, QCT_TPT = 65.79 °C, and QCT_GMS = 59.07 °C). The melting peaks of the solid lipids after loading QCT were not clearly different from those of the corresponding starting materials. The enthalpy of QCT_TLR, QCT_TPT, and QCT_GMS tended to be lower than that of the bulk solid lipids, changing from 180.55, 201.50 and 156.50 J/g to 86.11, 87.41, and 73.64 J/g, respectively. The deceasing enthalpies of SLNs compared with those of the bulk solid lipid are consistent with the finding of Fang et al. who reported that small particle sizes exhibited lower melting enthalpies than those of larger crystals [35]. 

### 3.4. Entrapment Efficiency and Drug Loading

The entrapment efficiency and drug loading for the QCT-loaded SLNs were calculated to evaluate the effects of solid lipids on the SLN properties. RMO:TLR 1:3 and RMO:TPT 1:3 were selected as representative formulations due to the particle sizes being smaller than 500 nm. The entrapment efficiency of QCT in RMO:TLR 1:3 was >60% whereas that of RMO:TPT 1:3 was 49.89 ± 3.51%. The drug loadings of all the studied formulations were low (~2% *w*/*w* due to drug expulsion from the crystalline matrix of the solid lipid) (Table 2) [36]. Despite the poor capacity of SLNs for loading QCT, we found that a QCT loading of only 1% was sufficient to achieve efficient anti-inflammatory effects in the formulated agent [37]. These results indicate that the type of solid lipid influenced the QCT entrapment. In the DSC thermogram, TPT exhibited a higher melting point than TLR. The high melting point was caused by high crystallinity, which reduced the entrapment efficiency and drug-loading capacity. Therefore, we concluded that a solid lipid with a low melting point was able to load a greater drug concentration than a solid lipid with a high melting point in the SLNs prepared by incorporating RMO.

### 3.5. Drug Release

**Figure 5:**
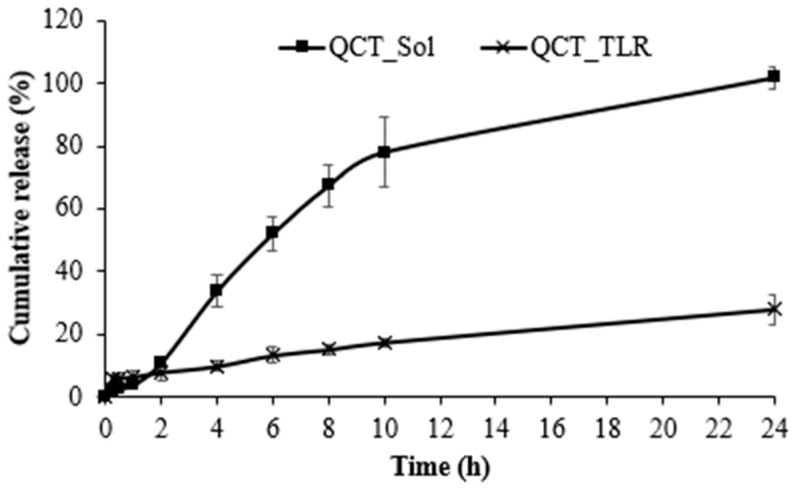
Release profile of QCT solution (QCT_Sol) and QCT_TLR.

A comparison of the cumulative release profile of QCT_SLN with that of QCT solution is shown in Figure 5. QCT_TLR is representative of QCT_SLNs because of the small particle size, high physical stability, and high entrapment efficiency. The QCT solution had a substantially faster release rate than SLN, with ~75% of the QCT dissolved in 10 h. Only 17% of the QCT was released from the SLNs within 10 h and 6% of the QCT was immediately released within the first 30 min, indicating a sustained release with a biphasic pattern [36]. During solidification, TLR mixed with RMO possibly crystallized, with an inner core comprising pure lipid first and then encapsulated QCT. The remaining QCT was formed around this lipid core in the shell region of the particle. QCT molecules located at the shell were immediately released within 30 min; ~6% of the QCT was continuously released over >24 h because of the QCT located in the lipid core [9,38]. The results suggest that TLR with RMO can be used to prolong the release of a carrier for lipophilic substances.

### 3.6. Cytotoxicity

**Figure 6:**
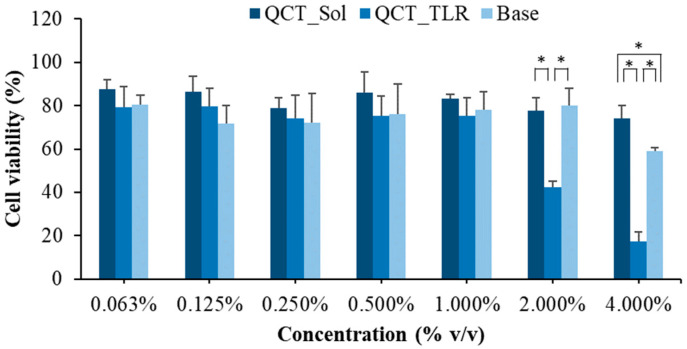
Percentage of cell viability after exposure to QCT_Sol (QCT solution in ethanol at the same concentration as those used in the SLNs), QCT_TLR, and base (10% *w*/*w* of CRH40 in water). * indicates significant difference (*p* < 0.05).

To assess the cytotoxicity of the QCT_SLN derived from the incorporation of RMO, an MTT assay with MRC-5 was performed. Similar to the drug-release test, the TLR formulation was selected as a representative SLN. As shown in Figure 6, the cytotoxicity of QCT_TLR had a tendency to increase with increasing concentration. In the samples with concentrations of <2% *v*/*v*, the cytotoxicities of the QCT solution, QCT_TLR, and base (CRH40 solutions) were not considerably different. At concentrations of ≥2%, the cytotoxicity was higher for the QCT_TLR than for the QCT and CRH40 solutions. The preparation of 4% *v*/*v* QCT_TLR was the most potent for arresting normal fibroblast cells, with a cytotoxicity of 17.24% ± 4.37%. The CRH40 solution exhibited high toxicity of 40.77%, whereas the QCT solution showed a slight cytotoxicity of 25.95% at a 4% *v*/*v* concentration. The high toxicity of QCT_TLR might have been due to a synergistic effect between CRH40 and QCT resulting from the enhanced permeability properties of the SLN [39]. Nevertheless, QCT_TLR at concentrations of <2% showed low cytotoxicity (percentage of cell viability > 75%) and could be used safely.

### 3.7. Molecular Dynamics Study

**Figure 7:**
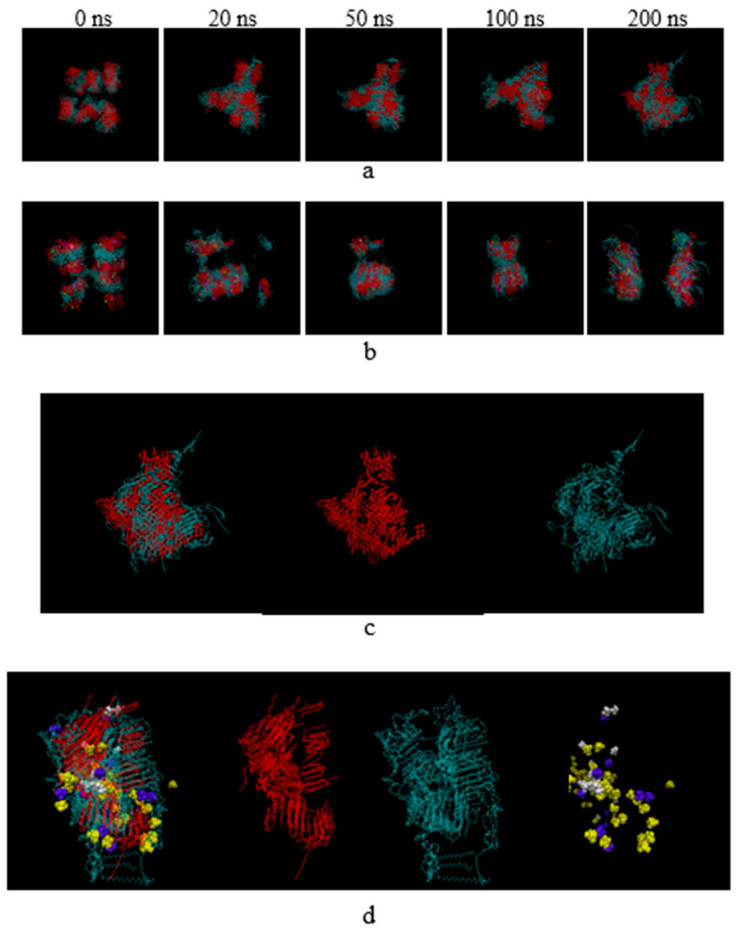
Computer simulation of SLNs without (**a**) and with RMO (**b**) during the simulation time from 0 to 200 ns. Self-assemblies of SLNs separated into each component without (**c**) and with (**d**) RMO at 200 ns. Red and blue represent TLR and CRH40, respectively, whereas white, violet, and yellow represent RMO including alpha-pinene, camphor, and eucalyptol, respectively.

Self-assembly is a crucial phenomenon for the formation of nanostructures involving the random aggregation of components to form a stable larger unit [40]. Herein, molecular dynamics simulations of SLN self-assembly were performed using Amber20 for 200 ns. The snapshots of the simulated self-assemblies of systems with and without RMO during 0–200 ns are shown in Figure 7. Water molecules were eliminated from the images to provide a clear view of the substrates in each system. As shown in Figure 7a, for the system without RMO, TLR aggregated to form large particles because of its high affinity for coherence. Conversely, for the system with RMO, an increase in the surface area or a decrease in particle size indicated the separation of assemblies (Figure 7b). Moreover, TLR and hydrophobic substances were located at the inside assemblies surrounding CRH40 (containing hydrophilic and hydrophobic parts), which play important roles in reducing interfacial tension between TLR and water (Figure 7c,d). RMO (eucalyptol, camphor, and alpha-pinene) was placed on the surface and inside of the SLN particle in the system with RMO (Figure 7d).

**Figure 8:**
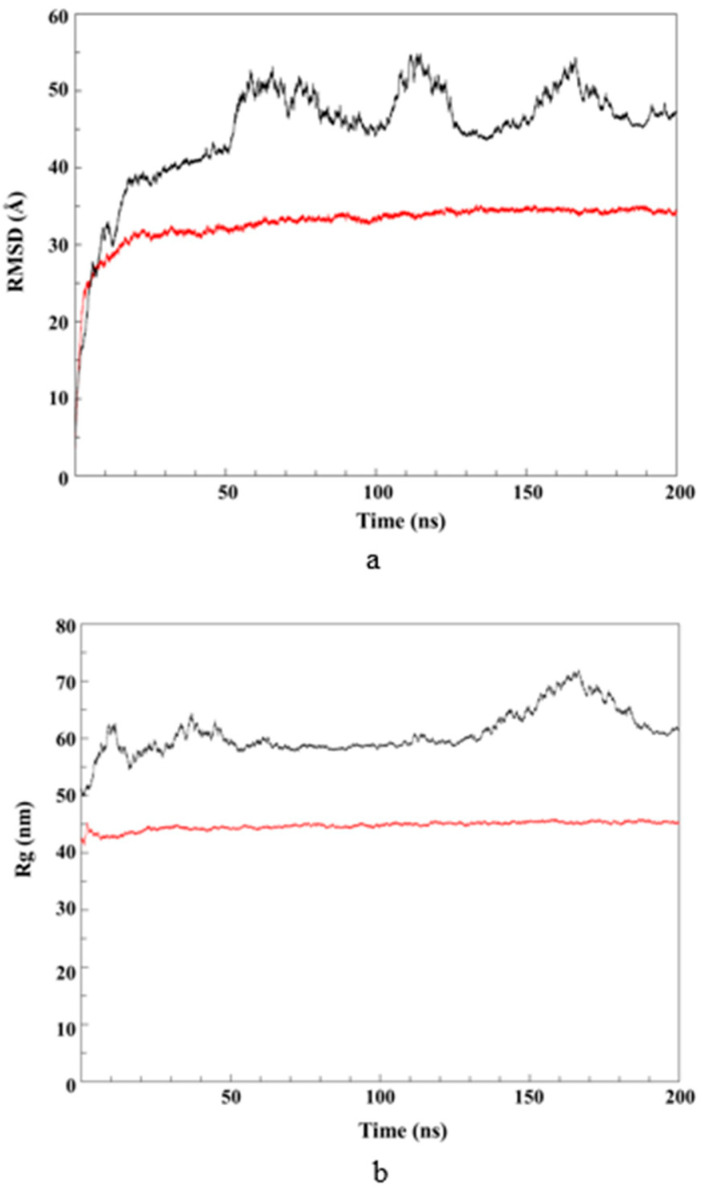
RMSD (**a**) and R_g_ (**b**) of assemblies in the SLNs without (shown in red) and with RMO (shown in black).

To thoroughly understand the structural evolution of SLNs during assembly formation, the RMSD of each assembly was calculated, as shown in Figure 8a. The assembly of the system without RMO reached equilibrium after 20 ns and remained constant at ~33 Å until 200 ns, indicating that the system without RMO aggregated within the first 20 ns and remained unchanged throughout the 200 ns. For the system with RMO, the RMSD fluctuated around 45 Å over the simulation time, indicating that there was a change in the structure of the system and the system did not aggregate into one large particle.

Additionally, R_g_ was calculated to determine the compactness and dynamic shape of systems. A low R_g_ value is indicative of a relatively compact polymer [41]. As shown in Figure 8b, the R_g_ value of the assembly without RMO was lower than that of the assembly with RMO, indicating a higher degree of structural compression [42]. The reason for this might have been that the RMO’s location within the TLR chain reduced the contact area between the TLR and water interface, leading to a lower interfacial tension for the TLR and water [43]. Therefore, the assembly with RMO was separated into a few small particles, whereas one particle with high compactness was observed for the assembly without RMO. The molecular dynamics simulations corresponded to the experimental results, supporting the idea that the presence of RMO, which contains a slightly water-soluble terpene, can help produce small-sized SLNs using a simple preparation process without applying an expensive tool.

## 4. Conclusions

SLNs loaded with QCT were successfully produced using a phase-inversion temperature method with RMO incorporation. This new method enabled the preparation of SLNs with small particle sizes, high physical stability, high entrapment efficiency, and good safety and did not require high energy or a complicated formulation. An RMO/TLR ratio of 1:3 with 10% *w*/*w* of CRH40 was the most suitable formulation. A dissolution test of the new carrier indicated sustained drug release with a biphasic pattern. A study of QCT_SLN self-assembly via molecular dynamics simulations provided a computer model that supported the idea that the presence of RMO can help produce small-sized SLNs more easily than a system without RMO. These findings support the potential of this method for the production of SLNs, especially for delivering low or poorly water-soluble bioactive substances.

## Figures and Tables

**Table 1:** Details of all the models used in the simulation processes.

System	SLN without RMO	SLN with RMO
Weight ratio of eucalyptol–camphor–alpha pinene–TLR–CRH40–water	0:0:0:1:1:8	0.116:0.029:0.028:0.750:1:8
Mole ratio of eucalyptol–camphor–alpha pinene–TLR–CRH40–water	0:0:0:16:4:4444	8:2:2:12:4:4444
Number of TLR molecules	96	96
Number of CRH40 molecules	24	24
Number of eucalyptol molecules	-	64
Number of camphor molecules	-	16
Number of alpha-pinene molecules	-	16
Number of water molecules	26,682	35,584
Total number of atoms in the system	102,414	135,472
Box size (x, y, z)	121, 108, 110	138, 107, 126

**Table 2:** Entrapment efficiency and drug loading.

SLN Formulation	Entrapment Efficiency (%)	Drug Loading (%)
1:3 TLR	63.41 ± 2.25	2.20 ± 0.04
1:3 TPT	49.89 ± 3.51	1.73 ± 0.03
